# Increasing harms for bingo players: digitisation, commercialisation and regulatory inadequacy: a multi-site case study

**DOI:** 10.1186/s12889-022-12954-y

**Published:** 2022-05-04

**Authors:** Kathleen Maltzahn, Mary Whiteside, Helen Lee, John Cox, Sarah MacLean

**Affiliations:** 1 Social Work and Social Policy, La Trobe University, Victoria, Australia; 2 Department of Social Inquiry, School of Humanities and Social Sciences, La Trobe University, Victoria, Australia; 3 School of Humanities and Social Sciences, La Trobe University, Victoria, Australia

**Keywords:** Gambling harms, Problem gambling, Bingo, Gambling regulation, Gaming, Public health

## Abstract

**Background:**

Bingo is often understood as a low-harm form of gambling. This view has been challenged by a growing body of literature identifying gambling harm to bingo players in a range of countries. In this study, we aimed to identify which conditions enabled, facilitated, intensified or mitigated gambling harm for bingo players in three populations in Victoria in the context of corporate, technological and regulatory changes.

**Methods:**

Our qualitative study investigated experiences of bingo-related gambling harm in three populations in Victoria, Australia where bingo was popular and structural disadvantage common: Indigenous people in the east, Pacific people in the state’s north and older people on low or fixed incomes in the capital. Data was generated through interviews with 53 bingo players and 13 stakeholders as well as 12 participant observations of bingo sessions.

**Results:**

We found that while bingo is overwhelmingly positive for many players, a minority of bingo players and their families experience notable harm. Harm was generated through traditional paper-based bingo games, new technologies such as tablet-based bingo and by the widespread tactic of placing bingo sessions in close proximity to harmful electronic gambling machines. Overall, the risk of harm to bingo players appears to be escalating due to commercial, technological and regulatory changes.

**Conclusions:**

These changes can be better managed by regulators: reforms are needed to safeguard bingo’s distinct character as a lower-risk form of gambling at a time when it, and its players, are under threat. Significantly, we found that harm to bingo players is intensified by factors external to gambling such as racialised poverty and adverse life events. Strategies that recognise these factors and grapple with gambling harm to bingo players are needed.

## Background

### Introduction

Bingo is a relatively simple game requiring little equipment that has become an enduring form of gambling in many countries. Early research on bingo explored why this modest game can become a constant and even compelling part of people’s lives [1]. More recently, there is growing academic and policy interest in the prevalence and impacts of gambling harm among bingo players [2]. We report here on a study of bingo playing across three sites in the Australian state of Victoria, drawing on the data to investigate types, causes and contexts of gambling harm to bingo players. As bingo players are disproportionately likely to be working class, women, older and/or Indigenous [1], our study sought to learn from the experiences of three geographically distinct groups of bingo players with some overlapping demographic characteristics: Indigenous people in regional towns in Victoria’s east, Pacific people in regional towns in the state’s north and older people on low or fixed incomes in the capital.

**Fig. 1:**
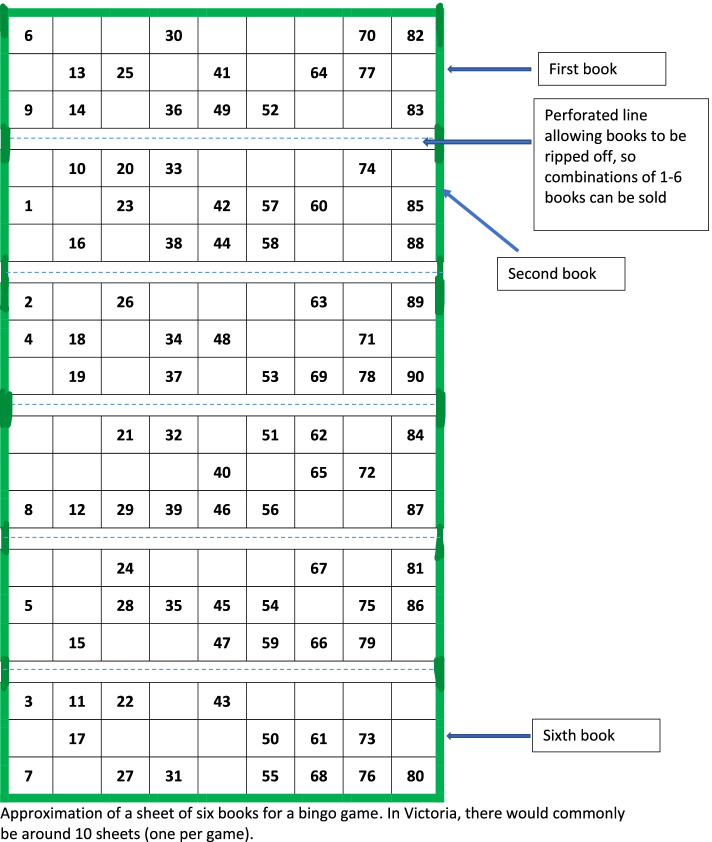
An approximation of a bingo book

Bingo is commonly played using a narrow strip of gridded paper displaying an incomplete set of numbers in ascending order, in Victoria typically between one and 90 (see fig. 1). Each grid corresponds to a game. Several sheets are combined to create a book, equipping a player to play several consecutive games. To enable players to play multiple books simultaneously, six grids are printed on one sheet of paper. Each grid is separated by a perforated line which allows books to be ripped off, so that players can request one or more books. Bingo books in Victoria are commercially printed. During a bingo session, a caller announces randomly generated numbers one-by-one, which players cross out, often using a thick felt pen made for bingo playing, monitoring as they play how close they are to crossing off all their numbers. The first player to correctly alert officials that all the numbers on their sheet have been called wins the game. As described above, participants can play multiple books simultaneously. More complex equipment has been introduced into the game in recent years. For example, new tablet-based bingo products, called personal electronic tablets (PETS) in Australia, can be programmed to automatically cross off numbers. Tablets beep when only one number is left, to prompt the player to pay attention. This means that players’ only role is to call out when all their numbers have been crossed off.

### What is gambling harm?

The Lancet recently described gambling as ‘a source of potentially serious and wide-ranging harms, affecting an individual’s health, wealth, and relationships’ that ‘can become a lifelong struggle to avoid relapse’ [3]. Such a contribution reflects an intensifying focus in gambling literature on harm and its forms, causes, consequences and reach [3–8]. Gambling harm is understood here as a continuum, with many people exposed to harm even while gambling in ways previously understood as low risk [9]. The burgeoning recognition of gambling harm has been both cause and consequence of intensifying calls for a public health approach to gambling [6]. In turn, increased recognition has led to a greater focus on defining and classifying gambling harm.

In a taxonomy of gambling harm built on earlier models (such as Abbott et al. [10]), Langham et al. identify seven types of harms: financial; relationship disruption, conflict or breakdown; emotional or psychological distress; decrements to health; cultural harm; reduced performance at work or study; and criminal activity [5]. These harms can be experienced by gamblers, their families and communities, and across three, sometimes overlapping, timeframes, described as points of crisis, as legacy harms that endure beyond crises and as life course or intergenerational harms. As a corrective to the dominant academic and policy focus on individual and psychological pathologies as the chief cause of gambling harm [9, 11], this work emphasises systemic and structural causal factors, including the actions and inactions of industry and government [3, 4], and the importance of cultural, economic, geographic, political and social contexts and determinants [9, 11]. While problem gambling research often aims to inform individual treatment options, gambling harm research emphasises public health and population-level strategies to prevent harm from occurring or intensifying.

### Gambling harm among bingo players

Engagement with questions of gambling harm in bingo literature has been influenced by several distinct characteristics of bingo players and bingo research, with research exploring bingo’s contribution to players’ lives (see, for example, Bedford [12] Bedford et al. [13]; Dixey & Talbot [14]; Maltzahn & Cox et al. [1]). In a context in which women were seen as morally suspect for gambling or stupid for enjoying bingo [15], sociological studies of bingo in particular have shown the many benefits bingo playing offered women, often in situations where their access to leisure and time with other women was severely impeded by lack of money, child-free time, transport and leisure opportunities or by cultural norms that constrained where women could go unaccompanied by men [14]. Bingo emerged as a site and activity where women felt connected, cognitively stimulated and temporarily released from stress or loneliness, as well as providing fun, excitement and the possibility of welcome cash. Nevertheless, harm has not been ignored in bingo literature. One example is the important Canadian study by Hewitt and Hodgson [16] of gambling among Indigenous people in Alberta, where bingo was the most common form of gambling. The study was commissioned by a First Nations organisation to inform prevention and treatment and identified high levels of significant gambling harm in the community with enduring and sometimes life-long implications. Hewitt and Hodgson’s research was groundbreaking in showing the social determinants of harm to bingo players, including links to trauma caused by colonial violence.

While bingo, particularly in its traditional form, has been understood at times as a low-risk form of gambling, and bingo players as largely free from negative impacts from their participation, more recent bingo literature has increasingly challenged these ideas. Maclure et al., for example, showed that for some women playing bingo in traditional halls, bingo was ‘a compulsion that can have painful personal consequences’ and which worsened their lack of economic control [17]. Other researchers have shown bingo-related financial losses and strain, consequent emotional and psychological distress and conflict for bingo players, their families and communities (see, for example, Wardle et al. [18]). As discussed above, bingo research consistently shows that such harm occurs in the context of the many positives of bingo playing, including its role in generating social connectedness, providing relief from sadness and strain and promising lighthearted fun as well as the chance of material gain. Maclure et al. [17] identified bingo’s inherent paradox: bingo can be both therapeutic and damaging, restorative and corrosive.

One theme of bingo research has been that players are variously and unfairly ignored, trivialised or denigrated by researchers, policy makers, media outlets and others, resulting in a failure to take bingo seriously as site for research or regulation [1]. This is pertinent in light of two areas of new evidence. First, that many gamblers, including bingo players, combine forms of gambling [18–21]. Second, that the likelihood or seriousness of harm may be influenced not only by the particular form of gambling undertaken, but also by the number of forms of gambling, and time and money spent by gamblers [22]. Ignoring bingo players leads to an incomplete understanding of who experiences gambling harm and how.

While bingo harm is often explored primarily in terms of individual losses, Bedford et al. [13] and Bedford [23] have expanded the criteria for assessing bingo’s impacts for individuals, communities and cultures. Bedford et al. [13] and Bedford [23] argued that bingo can be gainfully assessed against principles of fairness, which allows consideration of industrial impacts on bingo workers as well as players and their families. Second, Bedford identified the cultural and social contributions of bingo to collectives, such as building community cohesion and strengthening traditions of mutual aid, in turn highlighting the damage to these by regulatory pressure to standardise bingo as a generic gambling product. Such an analysis throws into relief the pivotal role of regulators and corporations in generating harm, illuminating the significance of political decisions rather than focusing on individual players’ actions. Complementing this capacious approach to conceptualising harm, Casey [24] built on earlier work (see for example Paarlberg et al. [25]) to highlight harm to charities which have become online bingo providers, arguing that they risk compromising their mandate and reputation. Additionally, exploring bingo in Brazil, Jobim and Williams [26] showed harm caused by money laundering and criminality in bingo businesses.

### Causes and contexts of harm

Notably, the literature shows that harm levels are not stable, but are influenced by cultural, economic, political and social conditions. These include interlinked structural disadvantages such as systemic racism and pre-existing levels of poverty and trauma, which are further shaped by, and shape, regulatory settings and available technology. Research in and by Indigenous communities, for example, has shown the symbiotic relationship between gambling harm and trauma, including trauma caused by colonial violence and other racism [16]. Feminist researchers have revealed the interplay between the impacts of bingo and cultural and economic constraints, such as being poor, on working-class and Indigenous women (for example, Bedford [23]; Fiske [27]). Illustrating the importance of regulatory settings, as explored above, Bedford [23] showed how regulations have chipped away at the distinct vernacular character of bingo. This homogenising process puts winning money at the heart of bingo, weakening the importance of collective and convivial elements of bingo and so creating conditions conducive to higher levels of harm. Equally importantly, a range of conditions, including strong social relations, can mitigate against gambling harm [28].

While regulatory decisions in wealthy English-speaking countries have tended to standardise and liberalise gambling, in contrast, in Brazil, regulators responded by first allowing and then criminalising bingo, providing an important political reminder that regulations that benefit gambling operators over individuals and communities are not inevitable but a political choice [26]. Finally, researchers have identified possible risks to bingo players due to new technologies including online bingo and terminal-based bingo: Harrigan et al. [29], for example, warned that new forms of electronic bingo incorporate higher-risk elements pioneered in Electronic Gambling Machines (EGM) technology and called for assessment of such innovation and Rockloff et al. suggest that new technologies risk inflating players’ illusion of control and likelihood of winning [30] (see [1] for further discussion of this). Evaluations of the real-world impact of such new technologies on bingo players have not yet been published. Our research explores these concerns and other issues and, in several areas, corroborates these studies.

The earlier focus of bingo studies on qualitative research that centres bingo players’ voices and views has diminished in recent years. Hence, qualitative player-focused research that examines questions such as harm and the impact of commercial, technological and regulatory changes on bingo is important. Additionally, although Bedford et al. [13] have conducted comparative research across different jurisdictions to examine the role of different regulatory approaches in shaping bingo, there has been little comparable attention in Australia, where bingo is very rarely researched.

### The Victorian context

There is limited research about the development of bingo in Victoria. Bingo, then known as housie-housie, was periodically banned up until the 1950s and was criminalised between 1954 and 1977: despite this, it was a popular pastime, particularly among women. When bingo was finally legalised, it was regulated as a fundraiser for sports and community clubs, with limits on the numbers and prices of tickets and a ban on paid staff and rolling jackpots. This changed through a series of regulatory changes in the mid-1980s, when bingo was explicitly professionaled through the introduction of bingo centres. While bingo centres were technically not-for-profit, in that profits still went to charitable or sporting organisations, they were run on business principles. However, when EGMs were introduced to Victoria in 1992, and bingo turnover and popularity plummeted, the state government loosened regulations. Aiming to make bingo more competitive, the price and number of tickets were increased, games were allowed on Sundays and paid staff were introduced [31]. Recent deregulation has allowed changes such as the introduction of rolling jackpots; combined with the introduction of PETs, prizes in some settings have increased significantly.

Bingo is offered in a range of forms and contexts, from small scale aged care homes and community-based bingo to clubs where it is provided in close proximity to EGMs, bingo centres with hundreds of players where bingo is the main form of gambling and, until recently, Melbourne’s sole casino, Crown Casino. Session prices range from free or low-cost to hundreds of dollars, and prizes from small monetary or material prizes to hundreds of thousands of dollars.

## Methods

Addressing these gaps, our article aims to investigate and compare the impact of bingo in the lives of people from three geographically discrete communities in Victoria, Australia where bingo is popular: Aboriginal people in Gippsland and East Gippsland in the south-east of the state, Pacific migrants in Mildura, in the north-west, and older people on low fixed incomes in the Victorian capital, Melbourne. As members of each of these communities face a range of sometimes overlapping forms of discrimination, exclusion and disadvantage, including racism, poverty and ageism, we aim to identify what conditions internal and external to gambling enable, facilitate, intensify or mitigate gambling harm for bingo players in these three communities.

We chose an instrumental multiple-case study approach and conducted our study in the Australian state of Victoria to enable us to engage with the complexity and diversity of bingo playing and players while at the same time examining one regulatory environment. Our approach was instrumental in that we wished to explore the issue of bingo, rather than specific groups themselves [32], multiple as we chose three populations and a case study approach as it facilitated, in Yin’s terms [33], an ‘up-close’, ‘in-depth’ understanding of bingo in a ‘real-world context’. We chose a qualitative approach as we wished to understand people’s experiences of harm and the meaning they made from their experiences, rather than to quantify or rank harms from gambling [34].

Informed by existing relationships with Aboriginal organisations in communities where bingo playing was popular, we were particularly interested in the role of bingo playing in populations that experience relative poverty, disadvantage and/or discrimination and the role of regulation in compounding or corroding these. Consequently, we chose three geographically distinct populations that would offer different perspectives on bingo playing and its context. Further, again informed by our partnerships with Aboriginal organisations, we wished to explore a wide range of impacts, including on communities, and so used the concept of gambling harms rather than the more psychologically and individually focused concept of gambling disorder.

We gathered data between September 2018 and October 2019 through individual, pair and group interviews with 53 bingo players, individual and pair interviews with 13 stakeholders and 12 participatory observation sessions of bingo games. Interviews with bingo players were up to an hour, with some stakeholder interviewers being up to 90 minutes, and were audio recorded. Field notes were taken after participant observations. We used criterion sampling [35], with criteria for interviews being that participants were either bingo players from one of the case study sites or an expert stakeholder with knowledge of bingo playing, other aspects of the case study sites or gambling and regulation in Victoria. To capture diversity, criteria for observation sites were that the venue had been identified by an interview participant and/or provided a distinct characteristic such as being in a regional area, a large bingo centre or a community-based game. An interview schedule with possible questions was developed by the research team, and provided a basis for interviewers. The interviews with members of the Aboriginal community were conducted by Aboriginal Research Fellow, [31] and those with the Pacific community largely by Mildura Pacific community member [31]. Bingo playing participants in Mildura and Gippsland/East Gippsland were recruited personally by interviewers; Melbourne participants responded to an advertisement in a seniors publication calling for interviewees. Interviews were conducted in a range of domestic, commercial and community settings. We did not record numbers of potential participants who chose not to participate. Stakeholder participants were approached by telephone, email and face-to-face. As they were community members, some of the bingo playing participants were known to Maltzahn & Thompson et al. [31], but not to other team members. One stakeholder was known to [removed for review] prior to recruitment. The remaining interviews were conducted by Maltzahn & Cox et al. [1]. Interviewers explained the purpose of the research as part of their introduction. Participant observations and data feedback were carried out by combinations of the authors listed here, six of whom are female and one of whom is male. Similar themes were raised consistently by participants towards the end of data collection at each site. The data was thematically analysed using NVivo (2018); coding was carried out by two team members. Analysis aimed to identify broad themes from the data on experiences of bingo playing as well as differences across communities and populations.

Our approach was informed by the Australian guidelines for researchers conducting research related to Aboriginal and Torres Strait Island people [36]. The guidelines call for research to ‘strengthen the research capabilities of Aboriginal and Torres Strait Islander people and their communities’ and ‘enhance the rights of Aboriginal and Torres Strait Islander Peoples as researchers, research partners, collaborators and participants in research’ [36]. One of several ways we did this was by appointing researchers from the communities concerned and reporting the findings back to communities in accessible ways, including through a short film by Aboriginal filmmaker, Caden Pearson (for more detail, see [31]). 1 We received ethical approval from the Latrobe University Human Research Ethics Committee (HEC18074 and HEC20260). Community participants are identified by a number plus G for Gippsland, M for Mildura, MAP for Melbourne; stakeholders are identified by S and a number, with those from the case study communities combining S and their area identifier.

We discuss the findings in the following categories: bingo players’ perceptions of gambling harm and causes and contexts of harm to bingo players, highlighting pricing and proximity to EGMs, new technologies, socio-economic factors and regulatory inadequacy.

## Results

### Bingo players’ perceptions of gambling harm

Only a minority of participants from our three case study populations said they had been harmed through bingo playing, however, for these people, the harms identified were, at times, significant. Interviewees described harm when playing traditional paper-based bingo, through exposure to EGMs (commonly called pokies in Australia) and from PETs. They also raised concerns about intensified harms caused by changes to bingo and some identified broader social and regulatory factors that increased harm. We start first by presenting the range of views about whether bingo could be harmful and then move to participants’ accounts of harm to bingo players and those around them.

Many participants across the three populations felt that bingo was overwhelmingly or only good: some felt that it was harm-free, describing inherent safety features such as time- and cost-limits and its cognitive stimulation and social rewards. Correspondingly, several participants explained that bingo was not generally seen as a form of gambling. Other participants saw harm levels as determined by external factors. For example, one stakeholder (S6), a gambling compliance expert, believed well-regulated bingo made a positive contribution to the community, saying ‘I think it is good for the community if it is properly regulated but it’s not being regulated at all’.

Using Langham et al.’s [5] classes of gambling harm to order our data, we found descriptions of financial, relationship, emotional and work/study-related harm that affected bingo players and their families, covering points of crisis, legacy and intergenerational harms. Financial harms included bingo players not being able to pay for basic living costs and pawning possessions such as phones to get cash to replace money spent on bingo or to play bingo.

Financial harms led to emotional strain. A Mildura participant (M7) described her heartbreak after coming home to her sleeping children, having lost at bingo, knowing her family was down to its last boxes of cereal and noodles. Stress was at times mitigated in Pacific and Aboriginal communities by strong family links as relatives would often help out: however, for some, assistance came with a sense of humiliation at having to ask, or see their parent ask, for help. Additionally, it could cause stress for those asked to give money, particularly when they could not afford to do so, another way that harm was felt by people beyond the gambler. Illustrating this, one Gippsland stakeholder explained that extended family members were impacted:

…because they’re the ones that have got to pick up the slack or provide for their young nieces or nephews, that haven’t got a school lunch or haven’t got the resources for what’s happening at schools, because mum or auntie have just spent it all at bingo (G1).

Financial and emotional strain also damaged relationships and fed conflict with partners, children and grandchildren. Several participants talked specifically about children bearing the brunt of adults’ frustrations. One Mildura woman in her 40s described her reaction to frequent bingo losses:

I ended up getting angry and my mood and behaviour was turning negative because I wasn’t winning. It changed how I would go about my daily activities. Like, for example, I would lose, come home and the kids would ask me something and I would already be mad and take it out on them by accident, and that’s not good. It’s not their fault I wasn’t winning (M6).

Work-related harms were raised, albeit infrequently, by participants. A small number described bingo players missing work commitments because they had played bingo until late or leaving work early to get to a bingo session.

### Causes and contexts of harm

As a form of gambling, bingo has inherent risk. However, the uneven levels of gambling harm for bingo players suggest that a range of causal factors facilitate gambling harm: the risk of significant harm is neither inevitable nor unchangeable. Our data highlighted both gambling-related and external causal factors, to which we now turn.

### Prices and proximity to EGMs

It was clear from participants that traditional paper-based bingo could cause harm and that bingo harm was not a new phenomenon, particularly for people with low incomes. This was underscored by interviewees’ accounts of going without essentials as children, before new technologies were introduced into bingo. The level of risk appeared to relate in part to the price of bingo, with more expensive forms of bingo being more harmful, with the exception of ‘free’ bingo, to which we will return below. Price was determined both by the price of an individual book and the number of books people bought. Some books cost as little as 50 cents, others AUD$8. While some players bought only one book, it was usual to buy several, and not uncommon to play six books. Bingo in our research sites typically ranged from less than $5 to up to $48 for six books. Particularly in the more expensive venues, players also commonly bought a stripped-down game of bingo called flyers, as well as instant lottery tickets, lucky envelopes and raffles, which increased the cost of a bingo session. Not surprisingly, those playing the low-cost versions were least likely to report harm. Attention to the cost of bingo in part explained the different patterns of harm amongst our three groups, with the Melbourne group of older people, who were more likely to play low-cost bingo and were in some cases wealthier, less likely to report harm.

The most common form of harm for bingo players was where bingo was offered in close proximity to EGM machines: bingo here appeared to be used to draw people into the venue with the expectation that they would then gamble on the EGMs. This practice was commonly used, whether in suburban and regional clubs or at Melbourne’s large and then politically powerful casino, Crown, where a Melbourne bingo player (MAP7) in her 70s described people coming ‘in bus loads.’ Both our interviews and participant observations confirmed widespread player movement from bingo games to EGMs. Some people used EGMs trying to recoup money spent at bingo and others spent their winnings on them, as one male Gippsland participant described:

Usually, to be totally honest with you, when I’ve won at bingo, I’ve normally gone straight to the pokies. [Laughs.] And tried to double it (G12).

Several participants knew bingo players whom they believed to be addicted to EGMs, describing significant associated harm. In several cases, participants saw such harm as resulting from a combination of conditions such as trauma or poverty with the contiguity of bingo to EGMs, as illustrated by a Melbourne participant in her 60s:

This particular friend of mine, … her son a few years ago committed suicide, because of the [gambling] debt he was in… [S]he also loved to play bingo. But we go and play bingo, after that, I say, 'come, let’s go'… and…when I’m there she’ll listen, but once she goes to the casino to play bingo, she will stay on. And she’ll tell me, 'oh dear, I lost $600 yesterday, and I lost so much' (MAP4).

#### New technologies

The second distinct context for harm described by participants was newer forms of electronic bingo, including, as described above, automated tablets (PETS) which require little intervention from players, and online bingo. While PETs were not available in all the venues we visited, where they were, they were very popular and many players combined a PET with paper-based games. While few players can play more than six paper-based books, PETs in Victoria have the technical capacity for around 200 concurrent games. Some venues set their own limits, commonly around 40. Participants described players paying hundreds of dollars for a single bingo session, and a special session at a popular Melbourne bingo centre that cost $800. High prices create a bigger prize pool, providing a substantial incentive to play, as one stakeholder working in an Aboriginal gambling program described:

…they’re spending over $100 a night [on PETs] there, you could do so much with that but they don’t think of that, they just think ‘oh but I’ve spent that, I’m going to win the ten grand’ and you’re like, ‘there’s 200 other people [who think] that are going to win it too’ (S11).

While there were fewer accounts of harm from online bingo, one older Gippsland man (G12) on a pension recounted accidentally spending $400 on online bingo when he had meant to spend $40, saying ‘I nearly had a heart attack… So after that I never went back’. Another Gippsland player (G2) said, ‘Probably every second person I know has either done it or is currently doing it and they’ll blow their credit…’. More commonly, however, players said they did not trust online bingo, found it boring or did not have the computer skills to play.

#### Regulatory inadequacy

Successive regulatory changes in Victoria have enabled more expensive bingo games, bigger bingo sessions and larger prizes [31]. These changes include abolishing bans on rolling jackpots and removing caps on the cost of books and numbers of players allowed per session. Bigger prizes appear to be a motivator for players to spend more at bingo, with some players not realising that there are more people vying for prizes and that less of the ticket money is distributed each game (for example, because the jackpots are rolling). Stakeholders also argued that the regulatory compliance regime was weaker than the past, with bingo operators able to operate with less government scrutiny. Stakeholders in particular argued that bingo was being regulated and managed by government as if it was still a small community concern, as one articulated:

You see community benefit statements [for EGMs]…they’re inadequate and they don’t paint a good picture, but I think there is a level of transparency that we've achieved in this state around poker machine losses that we simply don't get that kind of data on bingo or on lucky envelope machines. And I think that is really problematic given that these have become million-dollar businesses. They’re not, you know, taking hundred dollars a week in a friendly group of older people, you know, we’re talking about huge barns with hundreds of people, playing multiple games of bingo at once, staking significant amounts of money and some of them losing hundreds of dollars a night (S7).

The deregulation of bingo has created more pressure for bingo operators to adopt potentially more harmful approaches, such as PETs and high-cost bingo sessions. One industry stakeholder explained the market pressure to provide PETs:

When I first started here I would answer the phone and they’d say ‘what’s the jackpot tonight’, I’d be like ‘10,000 [dollars]’, [and they would reply] ‘oh great yep, alright, see you tonight’. If I answer the phone now and … I say ‘oh there’s a 5,000 and a 10,000 [dollars]’, [they reply] ‘is that all, only one 10,000, not two, not three’ and they don’t want to pay, they want it to be cheap but they want big [prizes] so the only way to do that is to have PET machines (S9).

### Socio-economic context

Regulatory changes interact with external factors. Racialised poverty and the impact of adverse life events were two of the external factors driving bingo-related gambling harm in our case study sites. The impetus to win money was greater among participants in the Gippsland and Mildura case study sites: both these case study communities have higher levels of poverty than the age pensioners in our Melbourne case study. For many Aboriginal participants, the immediate cause of poverty was the absence or low level of government benefits. Stakeholders from Aboriginal community-controlled organisations highlight the cumulative impact of low benefit payments across the community; more profoundly, poverty is a legacy of land and wage theft and ongoing colonial violence, discrimination and racism. In Mildura, many members of the Pacific community were employed in farm work that is casualised, low-paid, seasonal and hard, making it difficult to escape poverty. In the face of the family and community precarity and/or poverty that these factors cause for Indigenous and Pacific people, gambling can appear to be one of the few viable pathways to access large lump sums, as participants explained. Poverty shaped harm in two ways: it could make gambling compelling and also more harmful, as one Gippsland stakeholder from an Aboriginal organisation argued:

I think [bingo] has a more negative effect because, just as an Indigenous community … we have less income, we’re from poor socio-economic backgrounds, and I feel that half the people at the table that are working and can afford, and then there’s half that cannot afford to be there (G1).

While the amounts spent by bingo players have previously been seen as low risk because they are lower than in other forms of gambling, one stakeholder, who worked with a gamblers’ help service, pointed out that gambling costs are relative, saying that relatively small amounts could have a big impact for someone living in poverty:

Now the person who spends $10,000 [on gambling] would love to spend 80...but if you’re a pensioner spending 80, that’s probably a third of your weekly income (S13).

Adverse life events and stresses, at times resulting in trauma, were described by several participants. These included caring for elderly partners with dementia and other ill-health, post-surgical loss of cognitive capacity, raising grandchildren whose parents were in jail or struggling with drug addiction and family death. Here, bingo offered escape from grief, isolation and daily strains. Stakeholders from Aboriginal community-controlled organisations also described the trauma, isolation and disconnection from country experienced by members of the stolen generations (Aboriginal people who as children were unjustly removed from their parents by the government). Again, bingo and other forms of gambling provide an escape from stress and struggle. Particularly, but not only in the Aboriginal community, adverse life events were compounded by poverty. Participants described how the promise of relief from profound stress and/or poverty made bingo dangerously compelling for some.

While the majority of interviewees felt bingo was overwhelmingly positive in their own lives, gambling harm was a significant issue for a minority of players, the link between EGMs and bingo was seen as problematic by many participants and there was concern that harm would escalate as PETs and other product changes became more common. Participants also identified regulatory weaknesses and social injustices as contributing to harm. The varied nature of bingo’s impacts highlights the question of what elements, within the game or external to it, create harm. Our data suggests that protective factors that have made bingo relatively low risk are being eroded by commercialisation, technological changes and deregulation, and that risk of harm is intensified by factors external to bingo such as poverty and adverse life events.

## Discussion

Bingo is a straightforward and logical game. In contrast to the myriad of permutations for the order that numbers can be called out, the steps in the game are fixed and limited. Not so, however, are its consequences, as we have illustrated here. Our data across three case study sites provides support, as Maclure et al. [17] argued, that bingo is benign for many, while simultaneously compelling, disruptive and damaging for others. This study is one of the first to examine some of the mechanics of the infliction of gambling harm on bingo players.

The uneven distribution of gambling harm raises many questions about the sources of this tension and how bingo players are exposed to harm. In this study, we aimed to identify which conditions enabled, facilitated, intensified or mitigated gambling harm for bingo players in three populations in Victoria in the context of corporate, technological and regulatory changes. We were interested both in the manifestations of harm experienced by bingo players and their communities and in the contexts and causes of that harm.

We do not wish to dismiss the many and meaningful benefits of bingo, which we have explored in depth in other work [37]. Both prevalence data and qualitative studies demonstrate that the majority of bingo players play without adverse effects. In traditional paper-based bingo run in small local centres, gambling risk has been mitigated by other factors, such as bingo’s low and predictable cost. Here, its many benefits – from social connectedness to cognitive stimulation – frequently outweigh the risks. Many bingo players would arguably feel their life was diminished if they could not play bingo [37]. However, echoing research from other areas, our data indicates that changes to the game risk eroding these protective factors, reducing the benefits and exacerbating harms. Consequently, we find that bingo players in Victoria may be at greater risk of harm than previously. This is driven particularly by commercial, technical and regulatory changes that compound factors external to gambling that make gambling more dangerous for some people, such as poverty and racism, and adverse life events that cause stress and trauma. We identify several ways these changes intensify risk.

Our study illustrates the fact that bingo players frequently gamble in other ways, and that bingo is often combined with other forms of gambling such as raffles, lucky envelopes, and, of most concern, EGMs. This has been increasingly clear in bingo research and highlights the need to recognise that being a bingo player is not inherently protective, as bingo players can engage in other types of gambling that are higher-risk.

Our data further suggests that there is a relationship between the price of bingo, size of jackpots and levels of harm. In simple terms, the more players have to pay for play, the greater the potential for financial strain on them. Compounding this, big jackpots entice people to buy more books more often by generating hope that they can win big: in short, they make it seem worthwhile to gamble more as the potential rewards are higher than in the past. While unsurprisingly attractive to many players, bigger prizes centralise the financial benefit of bingo: rather than many players winning small amounts, which then offsets the costs of playing, bigger prizes benefit fewer people. Additionally, large prizes screen the fact that operators can retain large sums of money. Large jackpots are relatively new in Victoria and are possible because of regulatory changes and technological changes allowing linked and rolling jackpots and bigger crowds as well as new technologies such as PETs. For example, technological changes allow linked jackpots and games, where off-site callers are used and jackpots accumulate across multiple sites.

Our study is one of the first to explore the ways new technologies such as PETs are reshaping bingo, and the impact of this. In this, our study builds on work such as Harrigan et al. [29] and Rockloff et al. [30] that foreshadowed possible harm from technological developments in bingo. In showing the way bingo has been used to lure people to play EGMs, it also builds on work by Livingstone and Adams [38] that highlighted corporate forces’ use of local clubs, previously sites of low-harm ‘folk gambling’, to penetrate communities for extractive high-harm forms of gambling such as EGMs. Similarly, it takes Bedford’s [23] analysis of how regulators, through standardisation and other means, can damage bingo’s community-building role and shows this in action across three sites. For example, the bingo described by our respondents and that we observed was commonly played in commercial settings, in contrast to the church or community halls of previous times. Even where the clubs were technically not-for-profits, gambling was run as a business, prioritising profits over community benefit.

Together, these developments appear to transform an enjoyable, economical and low-risk outing with inbuilt protective factors into a higher-risk activity where for some accruing money becomes more important than any other aspect of the game. This undermines bingo’s place as a localised social activity where gambling, while fun, is not players’ sole motivation. Several stakeholders emphasised that this is a political and regulatory choice which could, and should, be changed.

The intensification of bingo as a form of gambling and the compounding impact of bingo players engaging in other types of gambling interact with factors external to gambling to generate harm. By exploring experiences of bingo in three communities with varying levels of structural disadvantage including exposure to systemic racism, our study highlights the way racism, poverty, stress and trauma interact with gambling harm. These often-preventable conditions appear both to make people more susceptible to gambling harm and to heighten and spread harm when it is incurred. This underscores the need to tackle factors external to gambling, such as racialised poverty, when seeking to prevent or alleviate gambling harm and to take such factors into account when assessing the impact of regulatory and other changes.

Our study provides an insight into bingo playing in disparate parts of Victoria that illustrates more generally the transformation of a vernacular form of low-harm gambling into a higher-risk extractive phenomenon and the preventable social injustices that expose some people to greater harm. In our study, three interlinked gambling-related changes were reshaping the game: commercialisation, new technologies and regulatory approaches. This in turn highlights government choices to allow or limit such changes.

### Policy implications

Our study provides support for the need for strategies to address gambling harm for bingo players, including by promoting fairness, protecting the benefits of bingo and preventing and constraining harm to bingo players. Such strategies should recognise that bingo players can accrue harm through traditional paper-based bingo as well as new technologies, and that bingo is implicated in harm as a pathway to EGM use as well as in itself a risky activity. Regulatory reform, including to manage the negative impacts of new technology as well as previous deregulation of the bingo industry, is an essential strategic tool. Such reforms should consider reintroducing limits on the cost of bingo and size of bingo gatherings and jackpots, separating bingo from EGMs and introducing caps on the allowable number and costs of PET games.

Factors external to gambling should be taken into account in two ways in devising and implementing such strategies. First, policy makers should ensure harm-reduction strategies respond to the specificities of different communities and bingo players, whether in terms of age, cultural background, socio-economic status or experiences of racism. Secondly, strategies that tackle factors external to gambling such as poverty reduction, trauma recovery and racism eradication should be acknowledged as legitimate ways to reduce the risks of gambling harm, and so should be included and resourced in gambling harm work. In recognising and responding to harm, policy makers must at the same time acknowledge, and seek to safeguard, the many positive aspects of bingo; ensuring that bingo players are at the heart of any policy processes will help such an undertaking. Additionally, consideration of gambling harm, including in legislation, should be expanded to include fairness.

### Limitations and future research

Our study had a number of limitations. Interviewees from the three case sites were self-selecting, and we do not claim that their experiences and views were representative of all members of the three identified populations. While, as previous research indicates, gender was clearly a factor shaping participants’ experience of bingo and gambling harm, it was not a strong theme in interviews and so we have not reflected here on the role of gender in shaping bingo playing and harm. Additionally, our study did not quantify the levels of harm or indeed of the benefits of bingo as experienced by participants, and so we cannot determine or compare the seriousness of harm experienced.

Further research would aid understanding of harm for bingo players. First, research exploring the impacts of PETs and other new technologies in bingo in other jurisdictions would provide additional information about these new developments. Second, studies investigating the interplay between gambling and external injustices such as racism, sexism, ageism and poverty would help broaden understandings of contexts for bingo playing and related harm. Third, in light of the limited research around strategies to minimise bingo-related gambling harm, investigation of regulations and other interventions to promote fairness, protect the benefits of bingo and prevent and constrain harm would contribute to both academic and policy discussions.

## Conclusion

Despite previously being seen as site of low gambling risk, and so offering little to interest those concerned with gambling harm, our study shows that bingo both generates harm and provides a fertile research and policy site for grappling with the complex causes and manifestations of gambling harm. Several factors make this so, including its uneven levels of harm, industry-level links between bingo and EGMs, the significant changes being wrought by commercial, technological and regulatory shifts, bingo’s distinct player groups and the significance of external factors such as structural racism and adverse life affects in shaping harm. Our portrayal of harm to bingo players in turn demands a response by regulators and other policy makers, highlighting the need for strategies to address gambling harm to bingo players.

## Data Availability

In accordance with ethical approval provided to conduct the project, data are not publicly available.

## Abbreviations

EGMs: Electronic Gambling Machines
PETs: Personal electronic tablets

## Footnotes

1 See https://www.youtube.com/watch?v=eIxpUYm7wEg&feature=youtu.be
