# Correction: Increasing harms for bingo players: digitisation, commercialisation and regulatory inadequacy: a multi-site case study

**DOI:** 10.1186/s12889-022-13418-z

**Published:** 2022-06-01

**Authors:** Kathleen Maltzahn, Mary Whiteside, Helen Lee, John Cox, Sarah MacLean

**Affiliations:** 1 Social Work and Social Policy, La Trobe University, Victoria, Australia; 2 Department of Social Inquiry, School of Humanities and Social Sciences, La Trobe University, Victoria, Australia; 3 School of Humanities and Social Sciences, La Trobe University, Victoria, Australia

**Correction: BMC Public Health 21, 664 (2021)**

**https://doi.org/10.1186/s12889-022-12954-y**

The original publication of this article [1] contained an a blinded sentence within the paper. The incorrect and correct information is listed below, the changes are shown in bold. The original article has been updated.

**Incorrect**

As they were community members, some of the bingo playing participants were known to Maltzahn & Thompson et al. **[31]**, but not to other team members. One stakeholder was known to **[removed for review]** prior to recruitment. The remaining interviews were conducted by Maltzahn & Cox et al. **[1].**

**Correct**

As they were community members, some of the bingo playing participants were known to Maltzahn & Thompson et al., but not to other team members. One stakeholder was known to **Maltzahn** prior to recruitment. The remaining interviews were conducted by Maltzahn & Cox et al.
